# Match-related physical performance in professional soccer: Position or player specific?

**DOI:** 10.1371/journal.pone.0256695

**Published:** 2021-09-10

**Authors:** Stefan Altmann, Leon Forcher, Ludwig Ruf, Adam Beavan, Timo Groß, Philipp Lussi, Alexander Woll, Sascha Härtel

**Affiliations:** 1 TSG ResearchLab gGmbH, Zuzenhausen, Germany; 2 Department for Performance Analysis, Institute of Sports and Sports Science, Karlsruhe Institute of Technology, Karlsruhe, Germany; 3 TSG 1899 Hoffenheim, Zuzenhausen, Germany; 4 Department for Social and Health Sciences in Sport, Institute of Sports and Sports Science, Karlsruhe Institute of Technology, Karlsruhe, Germany; University of Belgrade, SERBIA

## Abstract

The purpose of this study was to examine to what extent the physical match performance of professional soccer players is both position and player specific. First, official match data from the 2019/20 German Bundesliga season was used to search for players that met the inclusion criteria of playing a minimum of four entire matches in at least two different playing positions. Overall, 25 players met the criteria prior to the COVID-19 induced break, playing a minimum of eight matches. Second, the physical match performance of these players was analyzed separately for each position they played. The following four parameters were captured: total distance, high-intensity distance, sprinting distance, and accelerations. Third, the 25 players’ physical match performance data was then compared to normative data for each position they played to understand whether players adapted their physical performance (position dependent), or maintained their performance regardless of which position they were assigned to (position independent). When switching the position, the change in physical match performance of the respective players could be explained by 44–58% through the normative positional data. Moreover, there existed large individual differences in the way players adapted or maintained their performance when acting in different positions. Coaches and practitioners should be aware that some professional soccer players will likely incur differences in the composition of physical match performance when switching positions and therefore should pay special consideration for such differences in the training and recovery process of these players.

## Introduction

Soccer is characterized as an intermittent team-sport requiring professional players to cover total distances between 10 and 13 km per match [1, 2]. While the majority of the total distance occurs at lower intensities, 22–24% is spent at intensities above 15 km/h, 8–9% above 20 km/h, and 2–3% above 25 km/h. In addition, the players can perform between 600–650 accelerations during a match [3]. Hence, the typical match-related physical performance is reflected by a complex interaction of the aerobic and anaerobic energy systems [3].

Physical match performance has been shown to differ between playing positions [4]. The greatest total and high-intensity distance is commonly covered by central midfielders, wide defenders, and wide midfielders; while strikers and central defenders record lower distances [5, 6]. Regarding sprinting behavior, wide defenders and wide midfielders have been consistently reported to demonstrate the greatest sprinting distance, with similar values being obtained for forwards. Central midfielders demonstrate shorter total distances while sprinting, followed by central defenders [1, 5, 7–9]. In line with this, Mohr et al. [10] and Ingebrigtsen et al. [11] found that wide players exhibit greater sprinting distances than central players. Finally, as with sprinting, wide players seem to perform more accelerations than central players [10–12]. In sum, physical performance during matches differs between playing positions, both in the total distance itself as well as in its composition (i.e., high-intensity runs, sprints, accelerations).

Several studies have confirmed that a relationship exists between the players’ physical capacities (e.g., derived from endurance-, sprint-, and repeated-sprint tests) and their physical match performance (e.g., total distance, high-intensity distance, maximal sprinting speed) [13, 14]. That is, players with higher endurance or sprint capacities display higher total and high-intensity distances and reach higher maximal sprinting speeds during matches.

Therefore, it seems plausible that players in different positions also display distinct physical capacities. However, findings on position-specific single-sprint and repeated-sprint performance are inconclusive and no outfield position has been shown to constantly outperform other positions across several studies [15–17]. Moreover, recent research indicates that endurance capacities of professional outfield players are rather independent of their playing position [18, 19]. Consequently, it can be concluded that the above-described position-specific performance during matches is not always reflected by the players’ physical capacities.

Combined with the finding that there exists a high variability in physical match performance between players of the same position [8, 20, 21], a possible explanation for this observation might be that the physical match performance is not only dependent on the playing position but also to some extent on the individual players themselves [19]. In other words, while taking contextual factors such as team tactics, game location, opponent strength, congested period or match status into account that have all been shown to influence physical performance [22, 23], it might be further possible that some players always show a similar physical performance during matches, independent from the position they are instructed to play.

So far, only one study [24] has addressed this topic, showing a trend of players adapting their physical performance when switching playing positions. However, while reporting results on a group level, some limitations were not addressed in this study such as the inclusion of normative positional data from the same data set or the physical performance of individual players. Overcoming these limitations would allow for more meaningful conclusions to be drawn regarding whether the players’ physical match performance is position and player (in)dependent.

Therefore, the aim of this study was to examine to what extent the physical match performance of professional soccer players is attributed to being position and player specific by analyzing the individual data of players switching positions and normative positional data in relation to each other.

## Materials and methods

### Study design

In the present study, official match data from the 2019/2020 season of the German Bundesliga were used. To investigate to what extent the physical match performance of players is not only position but also player specific, first, all players that played at least in two different positions during the season were identified. Second, the physical match performance of these players (total distance, high-intensity distance, sprinting distance, number of accelerations) was analyzed separately for each position they played. Third, the obtained data were examined in relation to normative data for each position, thereby allowing the interpretation of whether the players in question either maintained or adapted their performance according to the normative positional data. The study was approved by the institutional review board of the Institute of Sports and Sports Science, Karlsruhe, Germany. Data were collected as a condition of employment in which player performance is routinely measured during match play. Therefore, informed consent by the players was not required for this study [25]. Nevertheless, to ensure team and player confidentiality, all data were anonymized prior to analysis.

### Subjects

Data were collected from the first 25 matchdays (i.e., before the COVID-19 induced break) during the 2019/2020 season of the German Bundesliga. To be included in the study, players must have completed at least four entire matches (full 90 min) in at least two different positions (i.e., in sum, a minimum of eight matches per player). A minimum of four matches per position was chosen to minimize the effect of contextual factors and to account for variability in physical performance [20–22, 26]. Moreover, only matches without a red card were included.

In total, 116 players were identified who completed at least one entire match in at least two different positions. However, only 25 players across 15 clubs met the inclusion criteria of at least four matches per position, thereby constituting the study sample. Collectively, from the 224 matches played in the study period, 163 matches were taken into account for the current study.

Normative data for each position were determined through all other players who were not included in the current study that also completed the full 90 min in one or more of the 163 matches in question, meaning that the 25 players included in the study sample did not also contribute to the normative data.

### Procedures

Each player of the study sample as well as those constituting the normative data were assigned to one of the following six outfield positions: central defender, wide defender, wing back, central midfielder, wide midfielder, forward. Regarding playing formation, in a system with four defenders (e.g., 4:4:2 or 4:2:3:1 system), the defensive players were coded as two central defenders and two wide defenders. Conversely, in a system with five defenders (e.g., 5:3:2 system), the defensive players were coded as three central defenders and two wing backs. For each player of the study sample, the main position, the secondary position, and, where applicable, the tertiary position was determined based on the number of matches played in the respective positions. Nevertheless, the order of position (main, secondary, tertiary) did not impact further analyses.

Furthermore, the physical match performance for each player and each position, respectively, was determined. The following four parameters were captured: total distance, high-intensity distance (17–23.99 km/h), sprinting distance (≥ 24 km/h), accelerations (positive acceleration values in each frame for ≥ 1.5 s). All definitions are based on the catalog of the German soccer league [27].

Both playing position and physical match performance data were derived from the official match data of the German Bundesliga. The latter was determined by means of a multiple-camera computerized tracking system (TRACAB, Chyron Hego, Melville, NY, USA) which has recently been validated [28].

### Statistical analysis

The data were analyzed using SPSS statistical software version 26.0 (SPSS, Inc., Chicago, IL). Mean values and standard deviations (SD) for each physical performance parameter were calculated regarding both the positional normative data and each player of the study sample for each position he played.

Possible differences in the normative data between playing positions were analyzed using one-way repeated measures analysis of variance (ANOVA) and subsequent pairwise comparisons with Bonferroni corrected p values. Cohen’s d effect sizes (ES) were calculated to quantify the magnitude of differences between positions. The ES was considered as small (0.2 ≤ ES < 0.5), moderate (0.5 ≤ ES < 0.8), and large (ES ≥ 0.8) [29].

To determine whether the players of the study sample either maintained or adapted their performance according to the normative positional data when playing in different positions, the data of the study sample and the normative data were examined in relation to each other. Specifically, the difference between the physical performance in the main position, the secondary position, and where applicable, the tertiary position was computed for each player of the study sample and examined by means of independent t-tests and ES. Moreover, the difference between the physical performance in the normative data for the position combinations that were evident in the study sample, e.g., central defender and wide defender, was computed. Lastly, Pearson’s product-moment correlations (r) with 95% confidence intervals (95% CI) were run between the positional difference in physical performance of the players in the study sample and the associated positional difference in the normative data. The magnitude of the correlation coefficient was considered as small (0.1 ≤ r < 0.3), moderate (0.3 ≤ r < 0.5), large (0.5 ≤ r < 0.7), very large (0.7 ≤ r < 0.9), and nearly perfect (r ≥ 0.9) [30]. The significance level for all statistical tests was set to 0.05.

## Results

Descriptive statistics (mean ± SD) of the normative positional data are reported in Table 1 and S1 Fig. The ANOVA revealed significant differences between playing positions for all physical performance parameters. While central midfielders showed both the largest total (11.66 ± 0.92 km, ES = 0.68–1.86) and high-intensity distance (1.57 ± 0.83 km, ES = 0.08–0.84) compared to all other positions, wide midfielders demonstrated the largest sprinting distance (0.42 ± 0.14 km, ES = 0.34–2.39), and wing backs performed the highest number of accelerations (512 ± 37, ES = 0.05–0.90) (see S1 Table).

**Table 1:** Normative data for total distance, high-intensity distance, sprinting distance, and number of accelerations separated by playing position. Results are presented as mean values ± SD.

	total distance [km]	high-intensity distance [km]	sprinting distance [km]	number of accelerations
*Whole sample (n = 1,964)*	10.87 ± 0.93	1.34 ± 0.56	0.27 ± 0.14	495 ± 45
*CD (n = 658)*	10.21 ± 0.64	1.04 ± 0.41	0.19 ± 0.08	484 ± 42
*WD (n = 244)*	10.75 ± 0.56	1.37 ± 0.23	0.36 ± 0.14	500 ± 39
*WB (n = 122)*	10.96 ± 0.55	1.48 ± 0.27	0.37 ± 0.11	512 ± 37
*CM (n = 538)*	11.66 ± 0.92	1.57 ± 0.83	0.24 ± 0.13	510 ± 44
*WM (n = 187)*	11.07 ± 0.73	1.51 ± 0.28	0.42 ± 0.14	494 ± 46
*FW (n = 215)*	10.86 ± 0.80	1.43 ± 0.30	0.34 ± 0.13	473 ± 47

SD—Standard deviation; CD—Central defender; WD—Wide defender; WB—Wing back; CM—Central midfielder; WM—Wide midfielder; FW—Forward.

Regarding the study sample, 23 players played in two different positions and two players in three different positions. In the latter case, all three positional comparisons were included for the two players in question (i.e., player 24/1, 24/2, 24/3, and player 25/1, 25/2, 25/3). The most common combinations of positions were wide defender and wing back (n = 9), central defender and wide defender (n = 6) as well as central midfielder and wide midfielder (n = 5).

**Fig 1:**
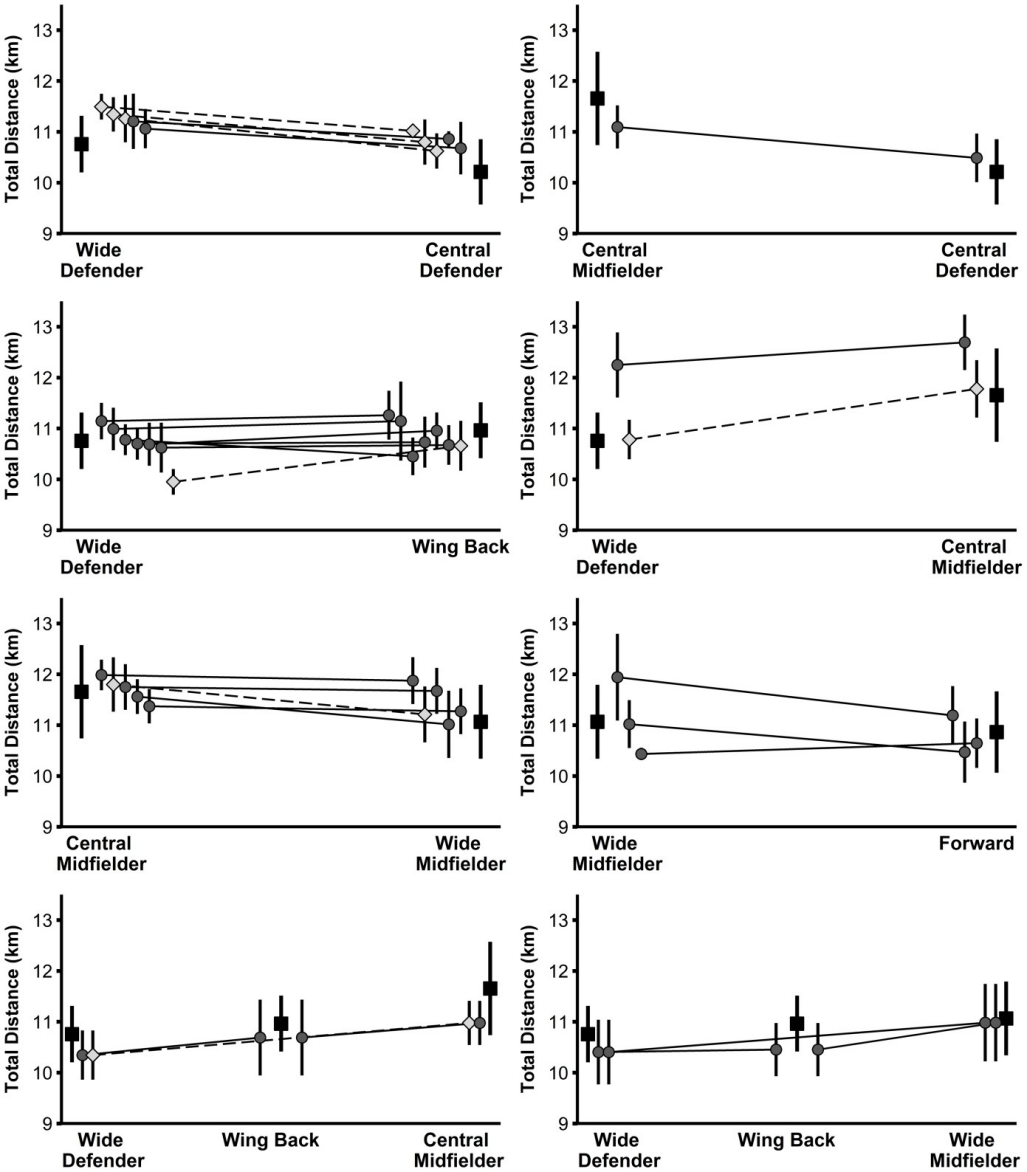
Total distance of players from the study sample (grey diamonds and circles) in relation to normative positional data (black squares). Data are presented as mean values ± SD. Light grey diamonds and dashed lines indicate significant differences in performance between the two positions for the respective player.

**Fig 2:**
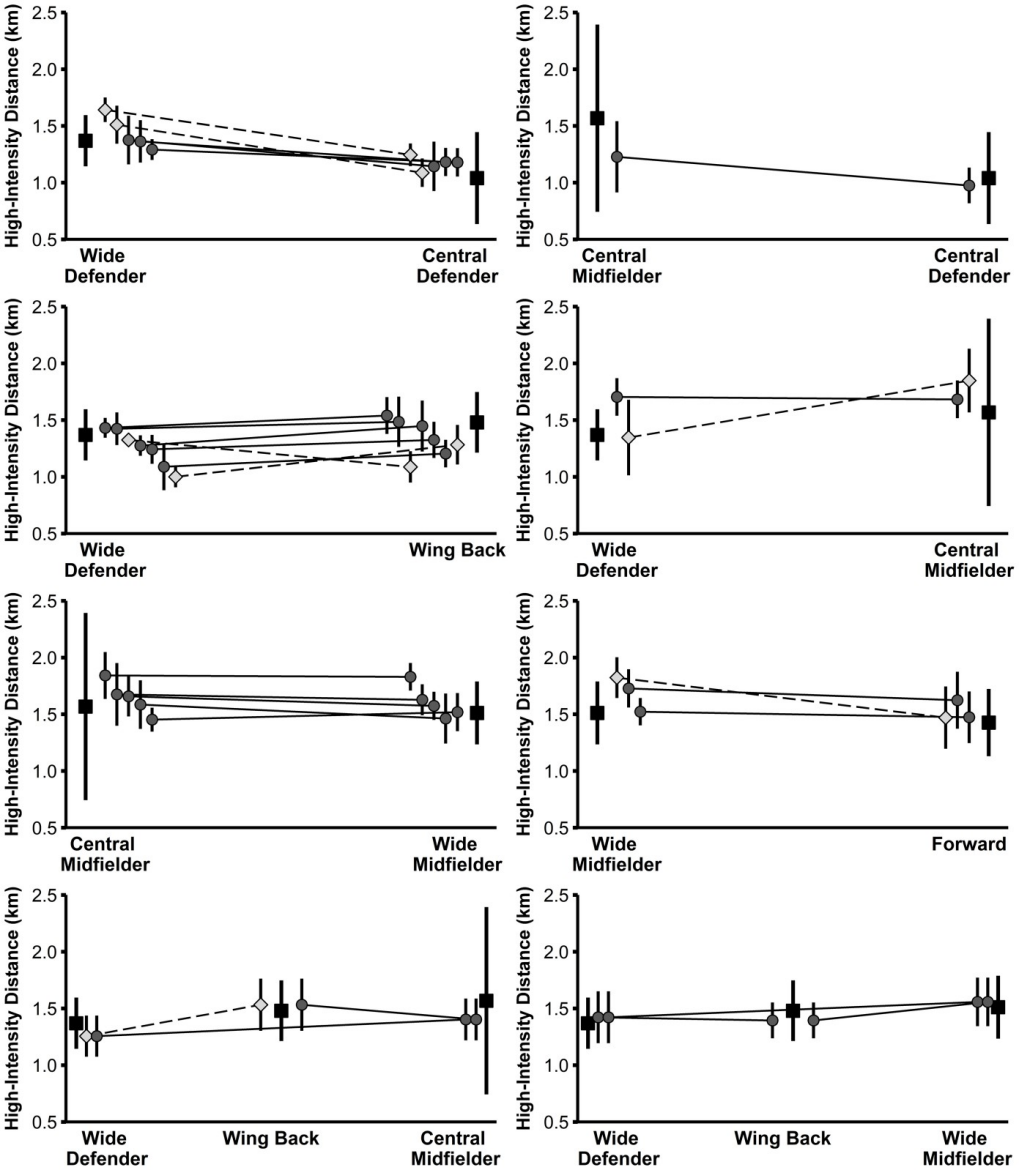
High-intensity distance of players from the study sample (grey diamonds and circles) in relation to normative positional data (black squares). Data are presented as mean values ± SD. Light grey diamonds and dashed lines indicate significant differences in performance between the two positions for the respective player.

**Fig 3:**
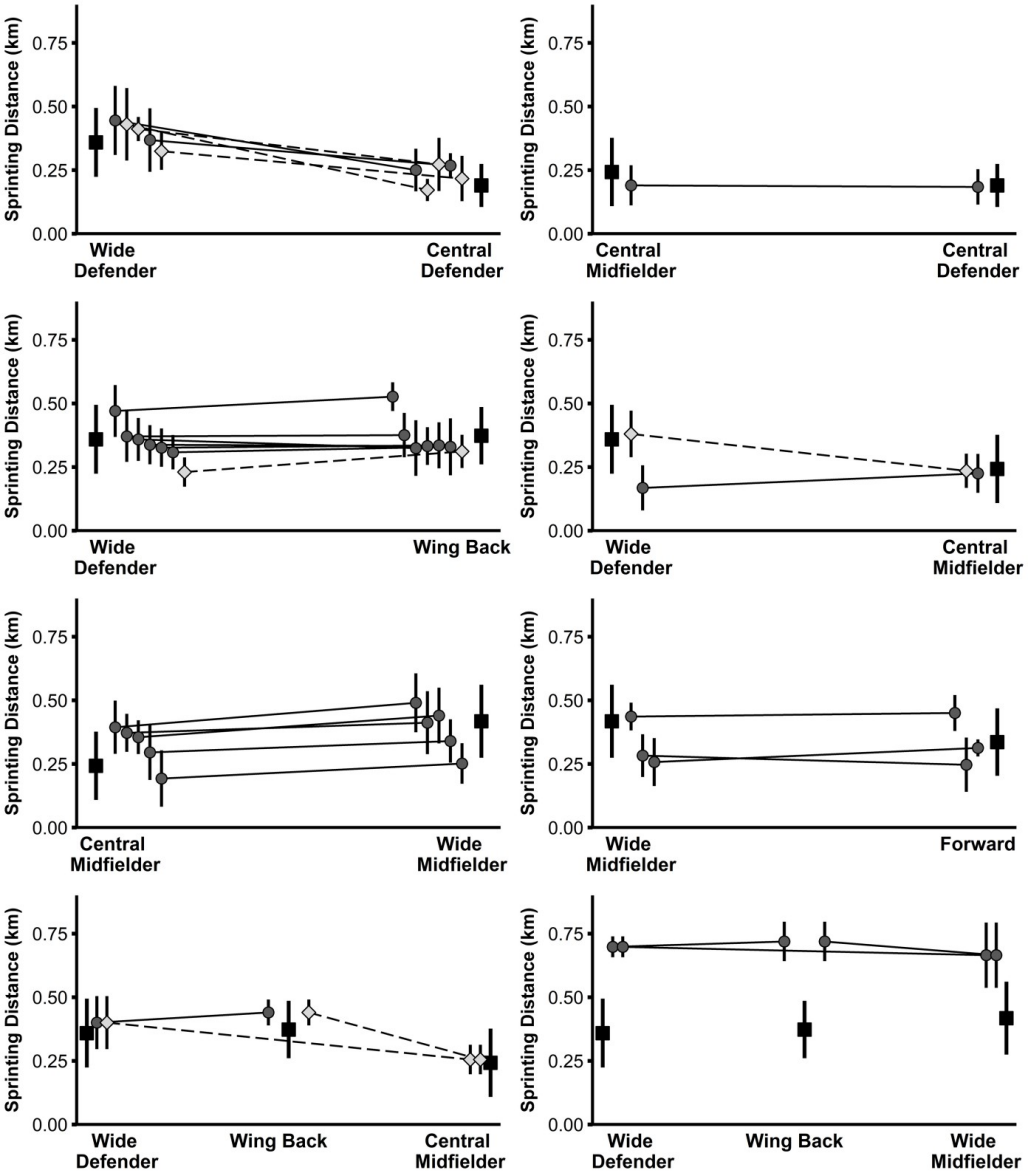
Sprinting distance of players from the study sample (grey diamonds and circles) in relation to normative positional data (black squares). Data are presented as mean values ± SD. Light grey diamonds and dashed lines indicate significant differences in performance between the two positions for the respective player.

**Fig 4:**
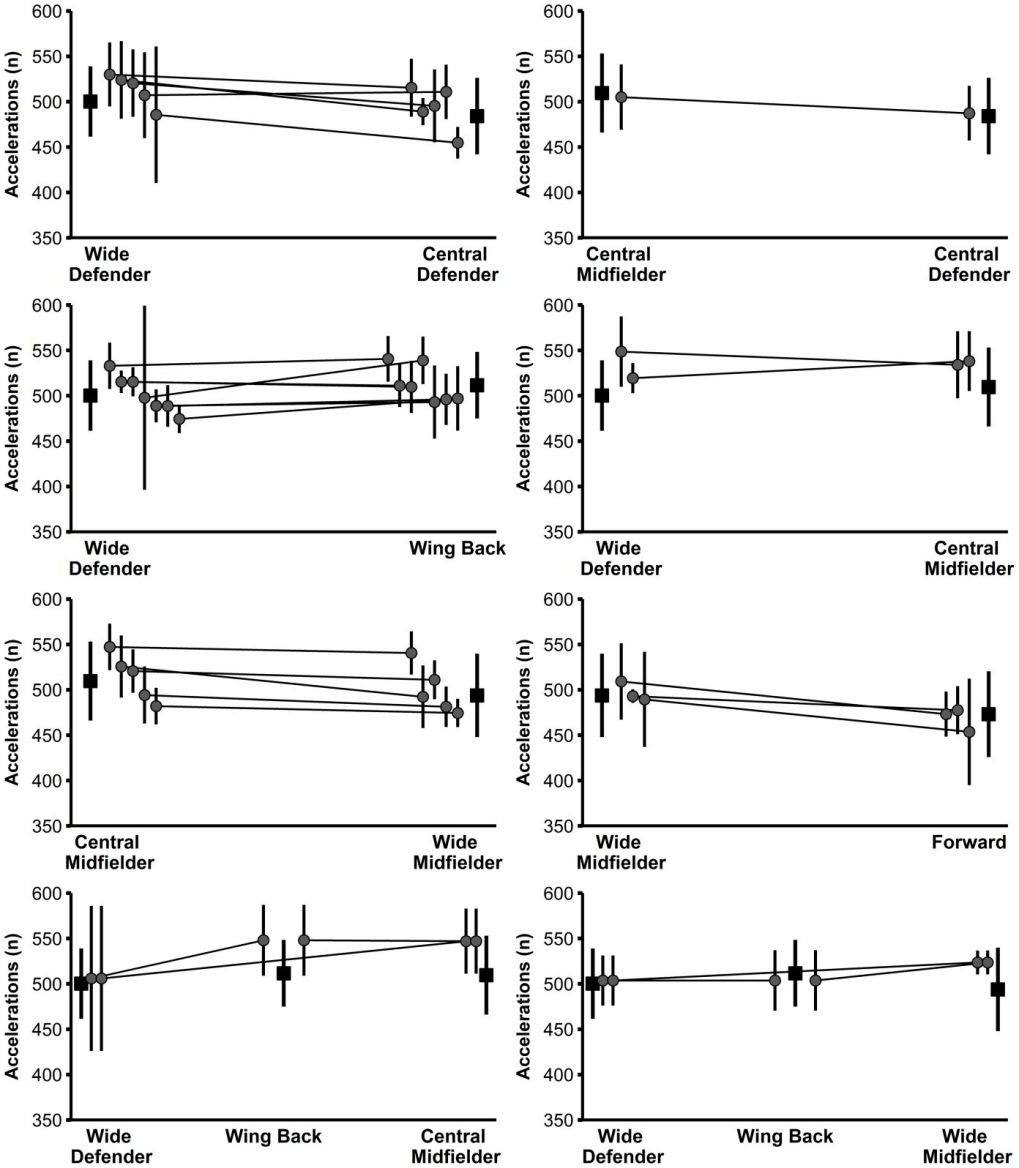
Number of accelerations of players from the study sample (grey diamonds and circles) in relation to normative positional data (black squares). Data are presented as mean values ± SD. Light grey diamonds and dashed lines indicate significant differences in performance between the two positions for the respective player.

Large to very large correlations (r = 0.66–0.76, r^2^ = 44–58%) were found between the positional difference in physical performance of the players in the study sample and the associated positional difference in the normative data (Table 2). Figs 1–4 illustrate the physical performance of each player of the study sample in relation to the positional normative data. Descriptive statistics (mean ± SD) and t-test results of each player of the study sample in relation to playing position are reported in S2 Table. Eight players clearly adapted their physical performance when changing the playing position supported by large observed ES differences between positions for at least three of the four performance parameters examined. Eleven players rather maintained their physical performance indicated from the large observed ES differences between positions for a maximum of one performance parameter. Nine players (representing 10 position combinations) displayed an inconsistent physical-performance pattern in relation to their playing positions demonstrated by large ES differences between positions for two performance parameters and trivial-to-moderate ES differences for two performance parameters). Moreover, large individual differences were observed in the way players behaved when acting in different positions.

**Table 2:** Pearson’s r (r^2^), 95% CI and p-values for correlations between the positional difference of the players in the study sample and the associated positional difference in the normative data for total distance, high-intensity distance, sprinting distance, and number of accelerations.

	total distance	high-intensity distance	sprinting distance	number of accelerations
Pearson’s r (r^2^)	0.76 (58%)	0.73 (53%)	0.76 (58%)	0.66 (44%)
95% CI	0.63–0.86	0.57–0.84	0.62–0.87	0.47–0.80
p-value	< 0.01	< 0.01	< 0.01	< 0.01

r^2^—Coefficient of determination; 95% CI—95% Confidence interval

## Discussion

The purpose of this study was to examine to what extent professional soccer players competing in the German Bundesliga adapted (position dependent), or maintained their performance regardless of which position they were assigned to (position independent).

The analysis of the normative data revealed pronounced positional differences regarding physical match performance serving as a basis for further analysis. Our results further indicate that changes in physical match performance of players can be explained by 44–58% by their playing positions while the remaining variance can be attributed to other factors such as the individual players themselves. In a similar fashion, there were pronounced individual differences in the way the players adapted or maintained their performance in relation to their positions.

Our findings on normative positional data in physical match performance support previous literature, while also adding several new insights. Regarding total and high-intensity distance, the highest values were achieved by central midfielders and wide midfielders, which is in line with previous research [5, 6]. Moreover, our results demonstrate that wide defenders (e.g., 4:4:2 or 4:2:3:1 system) displayed lower total and high-intensity distances compared to wing backs (e.g., 5:3:2 system), which is a new finding that highlights the necessity of distinguishing between these two positions. Wide midfielders, wing backs, and wide defenders followed by forwards demonstrated the greatest sprinting distance, while central midfielders and central defenders showed shorter distances while sprinting. These findings are generally supported by previous literature [1, 5, 7–9].

The last physical-performance parameter investigated in the present study is the number of accelerations. Here, wing backs, central midfielders, and wide defenders followed by wide midfielders accelerated most frequently. The high number of accelerations found in central midfielders contradicts recent studies [10–12] who reported wide players to perform more accelerations than central players. However, these studies included small sample sizes, used different definitions of accelerations, and were performed in different countries compared to our study, which could explain these discrepancies regarding central midfielders [31]. Besides, another interesting finding in relation to this parameter was that forwards accelerated least often of all positions, while central defenders demonstrated the lowest performance for the remaining parameters (i.e., total distance, high-intensity distance, sprinting distance). In summary, findings from our normative data reinforce that physical performance during matches differs between playing positions.

To investigate whether the players of the study sample either maintained or adapted their performance when playing in different positions, we analyzed the data of the study sample and the normative data in relation to each other. Correlation analyses revealed large to very large relationships between the positional difference in physical performance of the players in the study sample and the associated positional difference in the normative data. More specifically, changes in playing position explained 53–58% of the study sample’s variance in changes for total distance, high-intensity distance, and sprinting distance, and 44% for the number of accelerations. The remaining variance can be attributed to other factors such as the playing style of the individual players themselves.

Differences in the physical performance of each player of the study sample in relation to the normative data are clearly depicted within Figs 1–4 and S2 Table. From the study sample, eight players (players 3, 4, 11, 14, 17, 18, 19, and 24/1) clearly adjusted their physical performance according to the playing position. More specifically, one out of these eight players represented the position combination of wide defenders vs. wing backs, wide defenders vs. central midfielders, and wide midfielders vs. forwards, respectively. The remaining five players represented the combination of central defenders vs. wide defenders. Importantly, according to the normative data of the latter, wide defenders showed higher performance with large ES compared to central defenders for the three parameters total distance, high-intensity distance, and sprinting distance (see S1 Table). Therefore, distinct differences in the normative data might explain why some players from the study sample adjusted their physical performance according to the position. Our finding relating to the position combination of central defenders and wide defenders is supported by previous research [24] that also indicated large increases in performance when players switched from central to wide defender.

Another 11 players (player 1, 5, 8, 9, 12, 15, 20, 22, 23, 24/3, and 25/1) from the study sample maintained their physical performance irrespective of playing position. These players mainly represent position combinations with less distinct and less consistent differences according to the normative data (e.g., forwards vs. wide midfielders, wide defenders vs. wing backs, wing backs vs. central midfielders; see S1 Table). Therefore, it seems that the respective players from the study sample barely changed their performance as there was no need according to the positional normative data. Similarly, the behavior of players of the position combination of forwards and wide midfielders was comparable to that reported by Schuth et al. [24] who found only trivial to moderate ES differences within players interchanging between these two positions.

Lastly, nine players representing 10 position combinations (players 2, 6, 7, 10, 13, 16, 21, 24/2, 25/2, and 25/3) displayed a rather inconsistent physical-performance pattern in relation to their playing positions and, therefore, could not be attributed to one of the two aforementioned groups of players.

Besides this descriptive overview, large individual differences were observed in the way players behaved when acting in different positions. For instance, out of the three players representing the position combination wide defender and central midfielder, two players (players 2 and 24/1) decreased their sprinting distance by a large ES when playing as a central midfielder compared to playing as a wide defender. This change in sprinting performance is in accordance with the respective normative data. Conversely, the third player (player 10) representing this position combination increased his sprinting distance by a moderate ES, thereby contradicting the respective normative data. Another example with a similar pattern can be found in the position combination wide defender and wing back when looking at high-intensity distance. In agreement with the normative data (wing backs cover more high-intensity distance compared to wide defenders), out of nine players, four players (players 7, 14, 15, and 24/1) increased their performance by large ES and two players (players 22 and 23) by moderate ES when playing as a wing back, while 2 players (players 8 and 25/1) maintained their performance. By contrast, one player (player 6) of the same position combination decreased his high-intensity distance by a large ES in the wing-back position.

This is one of the first studies to investigate to what extent the physical performance during matches is not only position but also player specific. The importance of this topic is reflected by the fact that a total of 116 players completed at least one entire match in at least two different positions, leading to 178 single position combinations. Furthermore, considering the final study sample of 25 players, our results highlight that the playing position has a strong influence on the physical performance of players who act in two or more different positions, thereby supporting previous findings [24]. Albeit, there were pronounced individual differences in the way the players adapted or maintained their performance in relation to their positions.

While these individual differences can to some extent be explained by the individual playing style, another important factor that should be acknowledged in this regard is the variability of physical match performance [8, 20, 21, 32, 33]. In particular, it has been shown that variability differs between playing positions and the performance parameter in question [32, 33]. Therefore, especially on the individual level, it is complex to determine whether a real change in performance has occurred [32].

To account for this variability, we chose a minimum of four entire matches for a player to be included in the study sample. A drawback of this approach using a relatively high number of matches required is that it led to a relatively small sample size in which the playing positions were not evenly distributed. For example, only three players of the study sample acted as forwards, while 16 players acted as wide defenders. A possible explanation for this might be that offensive players are more likely to be substituted during a match compared to defensive players, thereby not fulfilling the inclusion criteria of completing the full 90 min [34]. Nevertheless, future studies including larger sample sizes and a more even distribution of positions are warranted to investigate whether our findings are generalizable. Moreover, such large-scale studies could also take contextual factors (e.g., team tactics, opponent strength) into account which were not considered in the present study [4]. Lastly, based on the large individual differences in the way players behaved when acting in different positions, it would be interesting to know which type of players (e.g., strong or weak physical capacities) adapt or maintain their performance.

## Conclusion

The findings of our study provide a number of potential practical applications, with the first relating to the connection between players adapting performance according to position and their physical capacities (e.g., sprinting and endurance performance). In particular, a change in playing position has a strong influence on the physical match performance of the players. Moreover, previous studies have shown that physical capacities are rather similar between players irrespective of their main playing position [15–19]. Therefore, players may experience different external and internal loads when changing between positions with commonly large performance differences, for example from central defender to wide defender. This change in load and the subsequent individual responses should be taken into consideration by coaches and practitioners in terms of the recovery process after matches. Second, the large individual differences observed highlight that physical match performance should not only be interpreted according to playing position but also to the individual players. Hence, coaches and practitioners should design training programs accounting for both the position(s) the players are supposed to act in and individuality.

## Supporting information

S1 Fig Normative data (mean values ± SD) for total distance, high-intensity distance, sprinting distance, and number of accelerations separated by playing position. (TIF)

S1 Table Mean difference, ANOVA, post-hoc test, and ES for total distance, high-intensity distance, sprinting distance, and number of accelerations between playing positions. (DOCX)

S2 Table Mean values ± SD, t-test results, and ES of each player of the study sample in relation to playing position for total distance, high-intensity distance, sprinting distance, and number of accelerations. (DOCX)
