# Supplementary figures and images for: Match-related physical performance in professional soccer: Position or player specific?
Source: PLoS One. 2021 Sep 10;16(9):e0256695. doi: 10.1371/journal.pone.0256695 (PMC8432651; doi:10.1371/journal.pone.0256695)

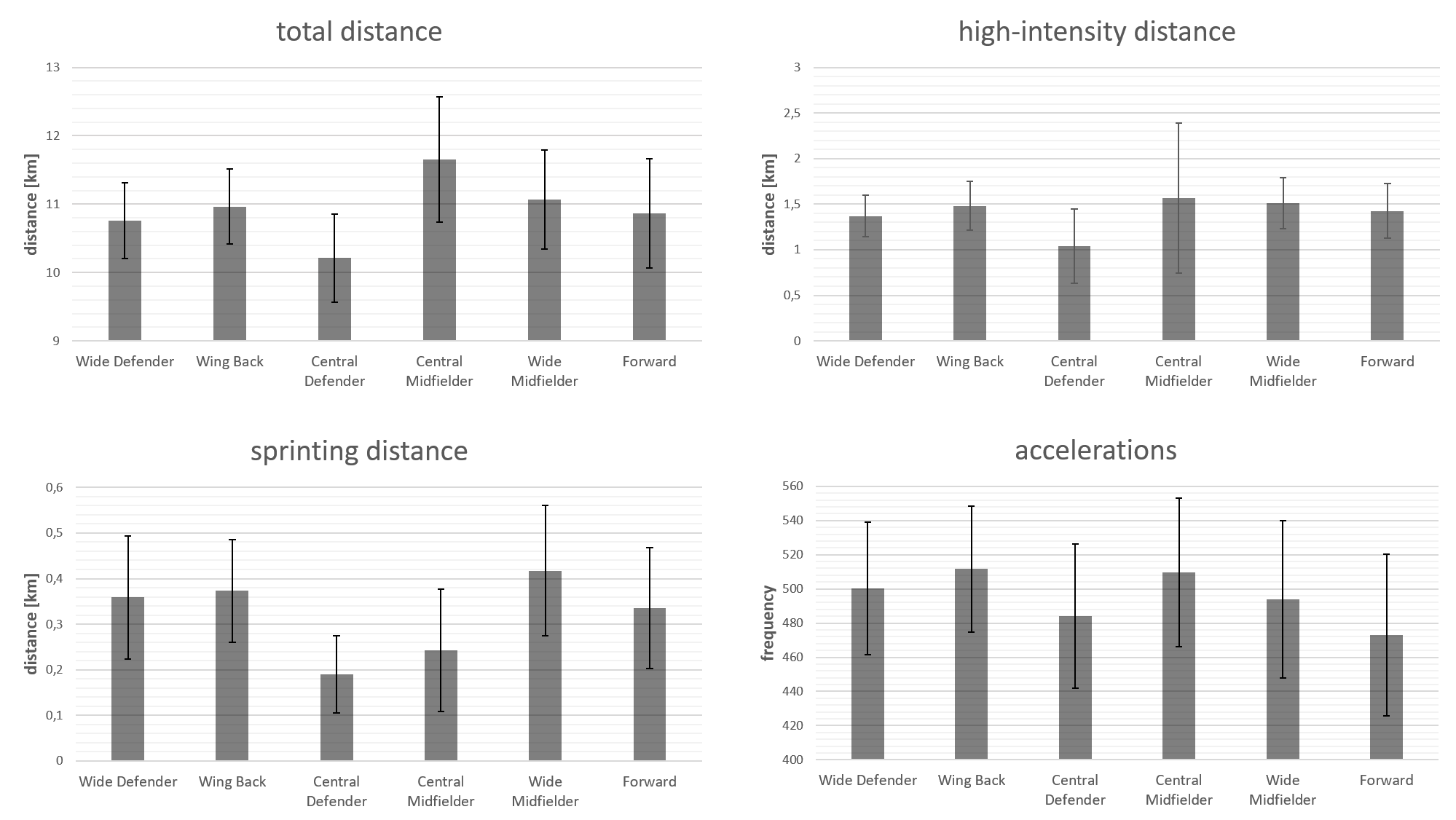

Supplement: S1 Fig — (TIF) [file pone.0256695.s001.tif]
