# Supplementary material for: Match-related physical performance in professional soccer: Position or player specific?
Source: PLoS One. 2021 Sep 10;16(9):e0256695. doi: 10.1371/journal.pone.0256695 (PMC8432651; doi:10.1371/journal.pone.0256695)
Supplement: S1 Table — (DOCX) [file pone.0256695.s002.docx]

**S1 Table.** Mean difference, ANOVA, Post-hoc test, and ES for total distance, high-intensity distance, sprinting distance, and number of accelerations between playing positions.

|  | **Mean difference (95% CI)** | **ANOVA** | **Post-hoc test** | **ES** |
| --- | --- | --- | --- | --- |
| **total distance [km]** |  |  |  |  |
| *CD vs. WD* | -0.55 (-0.42 – -0.67) | p < 0.01 | p < 0.01 | 0.87 |
| *CD vs. WB* | -0.75 (-0.59 – -0.92) |  | p < 0.01 | 1.20 |
| *CD vs. CM* | -1.44 (-1.58 – -1.31) |  | p < 0.01 | 1.86 |
| *CD vs. WM* | -0.85 (-1.03 – -0.68) |  | p < 0.01 | 1.30 |
| *CD vs. FW* | -0.65 (-0.83 – -0.47) |  | p < 0.01 | 0.95 |
| *WD vs. WB* | -0.21 (-0.39 – -0.03) |  | p = 0.01 | 0.38 |
| *WD vs. CM* | -0.90 (-1.05 – -0.74) |  | p < 0.01 | 1.10 |
| *WD vs. WM* | -0.31 (-0.50 – -0.12) |  | p < 0.01 | 0.50 |
| *WD vs. FW* | -0.11 (-0.30 – 0.09) |  | p = 0.81 | 0.16 |
| *WB vs. CM* | -0.69 (-0.88 – -0.50) |  | p < 0.01 | 0.81 |
| *WB vs. WM* | -0.10 (-0.32 – 0.11) |  | p = 0.93 | 0.17 |
| *WB vs. FW* | 0.10 (-0.12 – 0.32) |  | p = 0.94 | 0.14 |
| *CM vs. WM* | 0.59 (0.40 – 0.79) |  | p < 0.01 | 0.68 |
| *CM vs. FW* | 0.79 (0.59 – 1.00) |  | p < 0.01 | 0.90 |
| *WM vs. FW* | 0.20 (-0.02 – 0.43) |  | p = 0.12 | 0.27 |
| **high-intensity distance [km]** |  |  |  |  |
| *CD vs. WD* | -0.33 (-0.27 – -0.39) | p < 0.01 | p < 0.01 | 0.89 |
| *CD vs. WB* | -0.44 (-0.35 – -0.52) |  | p < 0.01 | 1.13 |
| *CD vs. CM* | -0.53 (-0.64 – -0.41) |  | p < 0.01 | 0.84 |
| *CD vs. WM* | -0.47 (-0.55 – -0.40) |  | p < 0.01 | 1.22 |
| *CD vs. FW* | -0.39 (-0.46 – -0.31) |  | p < 0.01 | 1.01 |
| *WD vs. WB* | -0.11 (-0.19 – -0.03) |  | p < 0.01 | 0.45 |
| *WD vs. CM* | -0.20 (-0.31 – -0.09) |  | p < 0.01 | 0.29 |
| *WD vs. WM* | -0.14 (-0.22 – -0.07) |  | p < 0.01 | 0.55 |
| *WD vs. FW* | -0.06 (-0.13 – 0.02) |  | p = 0.28 | 0.23 |
| *WB vs. CM* | -0.09 (-0.21 – 0.04) |  | p = 0.46 | 0.12 |
| *WB vs. WM* | -0.03 (-0.12 – 0.06) |  | p > 0.99 | 0.11 |
| *WB vs. FW* | 0.05 (-0.04 – 0.15) |  | p = 0.76 | 0.17 |
| *CM vs. WM* | 0.06 (-0.06 – 0.18) |  | p = 0.94 | 0.08 |
| *CM vs. FW* | 0.14 (0.02 – 0.26) |  | p < 0.01 | 0.19 |
| *WM vs. FW* | 0.08 (0.00 – 0.17) |  | p = 0.05 | 0.28 |
| **sprinting distance [km]** |  |  |  |  |
| *CD vs. WD* | -0.17 (-0.14 – -0.20) | p < 0.01 | p < 0.01 | 1.70 |
| *CD vs. WB* | -0.18 (-0.15 – -0.22) |  | p < 0.01 | 2.11 |
| *CD vs. CM* | -0.05 (-0.07 – -0.03) |  | p < 0.01 | 0.47 |
| *CD vs. WM* | -0.23 (-0.26 – -0.20) |  | p < 0.01 | 2.39 |
| *CD vs. FW* | -0.15 (-0.17 – -0.12) |  | p < 0.01 | 1.58 |
| *WD vs. WB* | -0.01 (-0.05 – 0.03) |  | p = 0.99 | 0.08 |
| *WD vs. CM* | 0.12 (0.09 – 0.15) |  | p < 0.01 | 0.90 |
| *WD vs. WM* | -0.06 (-0.10 – -0.02) |  | p < 0.01 | 0.34 |
| *WD vs. FW* | 0.02 (-0.01 – 0.06) |  | p = 0.63 | 0.15 |
| *WB vs. CM* | 0.13 (0.10 – 0.17) |  | p < 0.01 | 1.03 |
| *WB vs. WM* | -0.04 (-0.09 – 0.00) |  | p = 0.04 | 0.39 |
| *WB vs. FW* | 0.04 (0.00 – 0.08) |  | p = 0.09 | 0.24 |
| *CM vs. WM* | -0.18 (-0.21 – -0.14) |  | p < 0.01 | 1.36 |
| *CM vs. FW* | -0.09 (-0.12 – -0.06) |  | p < 0.01 | 0.77 |
| *WM vs. FW* | 0.08 (0.04 – 0.12) |  | p < 0.01 | 0.60 |
| **accelerations** |  |  |  |  |
| *CD vs. WD* | -16 (-7 – -26) | p < 0.01 | p < 0.01 | 0.39 |
| *CD vs. WB* | -28 (-15 – -40) |  | p < 0.01 | 0.68 |
| *CD vs. CM* | -25 (-33 – -18) |  | p < 0.01 | 0.61 |
| *CD vs. WM* | -10 (-20 – 1) |  | p = 0.94 | 0.23 |
| *CD vs. FW* | 11 (1 – 21) |  | p = 0.16 | 0.25 |
| *WD vs. WB* | -11 (-25 – 3) |  | p = 025 | 0.31 |
| *WD vs. CM* | -9 (-19 – 0) |  | p = 0.7 | 0.24 |
| *WD vs. WM* | 6 (-6 – 19) |  | p > 0.99 | 0.14 |
| *WD vs. FW* | 27 (15 – 39) |  | p < 0.01 | 0.63 |
| *WB vs. CM* | 2 (-11 – 15) |  | p > 0.99 | 0.05 |
| *WB vs. WM* | 18 (3 – 32) |  | p < 0.01 | 0.42 |
| *WB vs. FW* | 29 (24 – 53) |  | p < 0.01 | 0.90 |
| *CM vs. WM* | -16 (-26 – -5) |  | p < 0.01 | 0.36 |
| *CM vs. FW* | 37 (26 – 47) |  | p < 0.01 | 0.83 |
| *WM vs. FW* | 21 (8 – 33) |  | p < 0.01 | 0.45 |

ANOVA – Analysis of variance; ES – Effect size; 95% CI – 95% Confidence interval; CD – Central defender; WD – Wide defender; WB – Wing back; CM – Central midfielder; WM – Wide midfielder; FW – Forward
