# Supplementary material for: Match-related physical performance in professional soccer: Position or player specific?
Source: PLoS One. 2021 Sep 10;16(9):e0256695. doi: 10.1371/journal.pone.0256695 (PMC8432651; doi:10.1371/journal.pone.0256695)
Supplement: S2 Table — (DOCX) [file pone.0256695.s003.docx]

**S2 Table.** Mean values ± SD, t-test results, and ES of each player of the study sample in relation to playing position for total distance, high-intensity distance, sprinting distance, and number of accelerations.

|  | **Position 1** | **Position 2** | **t-test** | **ES** |
| --- | --- | --- | --- | --- |
| **Player 1 **** | FW (n = 6) | WM (n = 5) |  |  |
| *Total distance [km]* | 11.19 ± 0.58 | 11.94 ± 0.85 | p = 0.12 | 1.16 |
| *High-intensity distance [km]* | 1.62 ± 0.25 | 1.73 ± 0.17 | p = 0.45 | 0.56 |
| *Sprinting distance [km]* | 0.45 ± 0.07 | 0.44 ± 0.05 | p = 0.73 | 0.18 |
| *accelerations* | 473 ± 25 | 509 ± 42 | p = 0.11 | 1.18 |
|  |  |  |  |  |
| **Player 2 ***** | WD (n = 5) | CM (n = 12) |  |  |
| *Total distance [km]* | 12.25 ± 0.64 | 12.69 ± 0.55 | p = 0.17 | 0.81 |
| *High-intensity distance [km]* | 1.70 ± 0.17 | 1.68 ± 0.17 | p = 0.81 | 0.13 |
| *Sprinting distance [km]* | 0.38 ± 0.09 | 0.24 ± 0.07 | p < 0.01 | 1.96 |
| *accelerations* | 549 ± 39 | 534 ± 37 | p = 0.48 | 0.43 |
|  |  |  |  |  |
| **Player 3 *** | WD (n = 6) | CD (n = 6) |  |  |
| *Total distance [km]* | 11.21 ± 0.54 | 10.86 ± 0.15 | p = 0.16 | 0.97 |
| *High-intensity distance [km]* | 1.38 ± 0.21 | 1.14 ± 0.22 | p = 0.09 | 1.22 |
| *Sprinting distance [km]* | 0.43 ± 0.14 | 0.17 ± 0.04 | p < 0.01 | 2.77 |
| *accelerations* | 507 ± 47 | 511 ± 30 | p = 0.88 | 0.11 |
|  |  |  |  |  |
| **Player 4 *** | WD (n = 5) | CD (n = 4) |  |  |
| *Total distance [km]* | 11.49 ± 0.25 | 11.02 ± 0.12 | p = 0.01 | 2.60 |
| *High-intensity distance [km]* | 1.64 ± 0.11 | 1.25 ± 0.10 | p < 0.01 | 4.18 |
| *Sprinting distance [km]* | 0.37 ± 0.12 | 0.27 ± 0.05 | p = 0.18 | 1.18 |
| *accelerations* | 524 ± 43 | 489 ± 15 | p = 0.17 | 1.44 |
|  |  |  |  |  |
| **Player 5 **** | CM (n = 5) | WM (n = 5) |  |  |
| *Total distance [km]* | 11.37 ± 0.34 | 11.27 ± 0.45 | p = 0.70 | 0.28 |
| *High-intensity distance [km]* | 1.66 ± 0.18 | 1.57 ± 0.12 | p = 0.41 | 0.66 |
| *Sprinting distance [km]* | 0.37 ± 0.07 | 0.41 ± 0.12 | p = 0.55 | 0.46 |
| *accelerations* | 521 ± 24 | 511 ± 21 | p = 0.52 | 0.59 |
|  |  |  |  |  |
| **Player 6 ***** | WD (n = 6) | WB (n = 7) |  |  |
| *Total distance [km]* | 10.78 ± 0.30 | 10.45 ± 0.37 | p = 0.11 | 1.06 |
| *High-intensity distance [km]* | 1.33 ± 0.05 | 1.09 ± 0.14 | p < 0.01 | 2.40 |
| *Sprinting distance [km]* | 0.36 ± 0.08 | 0.32 ± 0.11 | p = 0.55 | 0.45 |
| *accelerations* | 515 ± 12 | 511 ± 24 | p = 0.71 | 0.22 |
|  |  |  |  |  |
| **Player 7 ***** | WD (n = 6) | WB (n = 8) |  |  |
| *Total distance [km]* | 10.69 ± 0.42 | 10.96 ± 0.36 | p = 0.23 | 0.76 |
| *High-intensity distance [km]* | 1.43 ± 0.09 | 1.54 ± 0.16 | p = 0.17 | 0.88 |
| *Sprinting distance [km]* | 0.23 ± 0.06 | 0.31 ± 0.07 | p = 0.03 | 1.31 |
| *accelerations* | 489 ± 23 | 496 ± 28 | p = 0.61 | 0.29 |
|  |  |  |  |  |
| **Player 8 **** | WD (n = 6) | WB (n = 8) |  |  |
| *Total distance [km]* | 10.7 ± 0.32 | 10.73 ± 0.50 | p = 0.91 | 0.07 |
| *High-intensity distance [km]* | 1.42 ± 0.14 | 1.49 ± 0.22 | p = 0.57 | 0.40 |
| *Sprinting distance [km]* | 0.47 ± 0.10 | 0.53 ± 0.06 | p = 0.21 | 0.82 |
| *accelerations* | 515 ± 16 | 510 ± 29 | p = 0.68 | 0.22 |
|  |  |  |  |  |
| **Player 9 **** | FW (n = 9) | WM(n = 4) |  |  |
| *Total distance [km]* | 10.64 ± 0.49 | 10.4 ± 0.12 | p = 0.25 | 0.62 |
| *High-intensity distance [km]* | 1.47 ± 0.22 | 1.52 ± 0.12 | p = 0.70 | 0.27 |
| *Sprinting distance [km]* | 0.25 ± 0.11 | 0.28 ± 0.08 | p = 0.57 | 0.32 |
| *accelerations* | 477 ± 26 | 493 ± 7 | p = 0.28 | 0.77 |
|  |  |  |  |  |
| **Player 10 ***** | WD (n = 5) | CM (n = 12) |  |  |
| *Total distance [km]* | 10.78 ± 0.39 | 11.78 ± 0.57 | p < 0.01 | 2.02 |
| *High-intensity distance [km]* | 1.35 ± 0.33 | 1.85 ± 0.28 | p = 0.02 | 1.81 |
| *Sprinting distance [km]* | 0.17 ± 0.09 | 0.23 ± 0.08 | p = 0.25 | 0.77 |
| *accelerations* | 519 ± 17 | 538 ± 33 | p = 0.20 | 0.68 |
|  |  |  |  |  |
| **Player 11 *** | WD (n = 9) | CD (n = 6) |  |  |
| *Total distance [km]* | 11.06 ± 0.39 | 10.68 ± 0.52 | p = 0.12 | 0.92 |
| *High-intensity distance [km]* | 1.36 ± 0.19 | 1.18 ± 0.12 | p = 0.06 | 1.16 |
| *Sprinting distance [km]* | 0.32 ± 0.07 | 0.22 ± 0.09 | p = 0.02 | 1.54 |
| *accelerations* | 502 ± 37 | 495 ± 40 | p = 0.24 | 0.20 |
|  |  |  |  |  |
| **Player 12 **** | CM (n = 5) | WM (n = 7) | p = 0.78 | 0.19 |
| *Total distance [km]* | 11.75 ± 0.45 | 11.67 ± 0.45 | p = 0.90 | 0.07 |
| *High-intensity distance [km]* | 1.84 ± 0.21 | 1.83 ± 0.12 | p = 0.17 | 0.97 |
| *Sprinting distance [km]* | 0.39 ± 0.10 | 0.49 ± 0.12 | p = 0.42 | 0.55 |
| *accelerations* | 494 ± 31 | 481 ± 22 | p = 0.78 | 0.19 |
|  |  |  |  |  |
| **Player 13 ***** | CM (n = 10) | WM (n = 4) |  |  |
| *Total distance [km]* | 11.56 ± 0.34 | 11.02 ± 0.66 | p = 0.06 | 1.32 |
| *High-intensity distance [km]* | 1.68 ± 0.28 | 1.63 ± 0.14 | p = 0.75 | 0.21 |
| *Sprinting distance [km]* | 0.36 ± 0.07 | 0.44 ± 0.11 | p = 0.10 | 1.06 |
| *accelerations* | 482 ± 20 | 475 ± 16 | p = 0.5 | 0.40 |
|  |  |  |  |  |
| **Player 14 *** | WD (n = 5) | WB (n = 11) |  |  |
| *Total distance [km]* | 9.95 ± 0.25 | 10.66 ± 0.49 | p < 0.01 | 1.74 |
| *High-intensity distance [km]* | 1.00 ± 0.09 | 1.28 ± 0.17 | p < 0.01 | 1.98 |
| *Sprinting distance [km]* | 0.31 ± 0.07 | 0.33 ± 0.11 | p = 0.71 | 0.21 |
| *accelerations* | 474 ± 16 | 497 ± 35 | p = 0.20 | 0.97 |
|  |  |  |  |  |
| **Player 15 **** | WD (n = 4) | WB (n = 14) |  |  |
| *Total distance [km]* | 11.15 ± 0.36 | 11.26 ± 0.48 | p = 0.67 | 0.25 |
| *High-intensity distance [km]* | 1.28 ± 0.09 | 1.45 ± 0.22 | p = 0.16 | 0.89 |
| *Sprinting distance [km]* | 0.34 ± 0.08 | 0.33 ± 0.07 | p = 0.91 | 0.15 |
| *accelerations* | 533 ± 26 | 540 ± 25 | p = 0.62 | 0.29 |
|  |  |  |  |  |
| **Player 16 ***** | CD (n = 15) | CM (n = 4) |  |  |
| *Total distance [km]* | 10.49 ± 0.48 | 11.10 ± 0.42 | p = 0.03 | 1.37 |
| *High-intensity distance [km]* | 0.97 ± 0.16 | 1.23 ± 0.31 | p = 0.03 | 1.41 |
| *Sprinting distance [km]* | 0.18 ± 0.07 | 0.19 ± 0.08 | p = 0.90 | 0.15 |
| *accelerations* | 487 ± 30 | 505 ± 36 | p = 0.33 | 0.61 |
|  |  |  |  |  |
| **Player 17 *** | FW (n = 7) | WM (n = 7) |  |  |
| *Total distance [km]* | 10.47 ± 0.60 | 11.02 ± 0.47 | p = 0.81 | 1.10 |
| *High-intensity distance [km]* | 1.47 ± 0.27 | 1.82 ± 0.18 | p = 0.02 | 1.65 |
| *Sprinting distance [km]* | 0.31 ± 0.03 | 0.26 ± 0.09 | p = 0.25 | 0.81 |
| *accelerations* | 454 ± 59 | 489 ± 52 | p = 0.17 | 0.68 |
|  |  |  |  |  |
| **Player 18 *** | WD (n = 16) | CD (n = 4) |  |  |
| *Total distance [km]* | 11.26 ± 0.47 | 10.62 ± 0.35 | p = 0.02 | 1.49 |
| *High-intensity distance [km]* | 1.51 ± 0.17 | 1.09 ± 0.13 | p < 0.01 | 2.70 |
| *Sprinting distance [km]* | 0.45 ± 0.14 | 0.25 ± 0.08 | p = 0.14 | 1.60 |
| *accelerations* | 486 ± 75 | 455 ± 17 | p = 0.44 | 0.47 |
|  |  |  |  |  |
| **Player 19 *** | WD (n = 7) | CD (n = 9) |  |  |
| *Total distance [km]* | 11.34 ± 0.34 | 10.8 ± 0.44 | p = 0.02 | 1.44 |
| *High-intensity distance [km]* | 1.29 ± 0.09 | 1.18 ± 0.12 | p = 0.07 | 1.09 |
| *Sprinting distance [km]* | 0.41 ± 0.05 | 0.27 ± 0.10 | p < 0.01 | 1.82 |
| *accelerations* | 530 ± 35 | 515 ± 32 | p = 0.40 | 0.48 |
|  |  |  |  |  |
| **Player 20 **** | CM (n = 4) | WM (n = 5) |  |  |
| *Total distance [km]* | 11.99 ± 0.30 | 11.87 ± 0.46 | p = 0.68 | 0.34 |
| *High-intensity distance [km]* | 1.45 ± 0.11 | 1.52 ± 0.17 | p = 0.50 | 0.54 |
| *Sprinting distance [km]* | 0.19 ± 0.11 | 0.25 ± 0.08 | p = 0.33 | 0.72 |
| *accelerations* | 547 ± 26 | 541 ± 24 | p = 0.69 | 0.27 |
|  |  |  |  |  |
| **Player 21 ***** | CM (n = 8) | WM (n = 8) |  |  |
| *Total distance [km]* | 11.80 ± 0.53 | 11.21 ± 0.55 | p = 0.05 | 1.17 |
| *High-intensity distance [km]* | 1.59 ± 0.21 | 1.46 ± 0.22 | p = 0.28 | 0.65 |
| *Sprinting distance [km]* | 0.30 ± 0.11 | 0.34 ± 0.03 | p = 0.37 | 0.53 |
| *accelerations* | 526 ± 34 | 492 ± 35 | p = 0.07 | 1.05 |
|  |  |  |  |  |
| **Player 22 **** | WD (n = 6) | WB (n = 15) |  |  |
| *Total distance [km]* | 10.62 ± 0.49 | 10.68 ± 0.39 | p = 0.80 | 0.15 |
| *High-intensity distance [km]* | 1.09 ± 0.21 | 1.20 ± 0.12 | p = 0.12 | 0.78 |
| *Sprinting distance [km]* | 0.37 ± 0.10 | 0.38 ± 0.09 | p = 0.81 | 0.11 |
| *accelerations* | 489 ± 18 | 493 ± 40 | p = 0.90 | 0.12 |
|  |  |  |  |  |
| **Player 23 **** | WD (n = 10) | WB (n = 4) |  |  |
| *Total distance [km]* | 10.99 ± 0.42 | 11.15 ± 0.78 | p = 0.63 | 0.32 |
| *High-intensity distance [km]* | 1.24 ± 0.13 | 1.33 ± 0.16 | p = 0.32 | 0.70 |
| *Sprinting distance [km]* | 0.33 ± 0.08 | 0.34 ± 0.09 | p = 0.86 | 0.13 |
| *accelerations* | 498 ± 102 | 539 ± 26 | p = 0.45 | 0.50 |
|  |  |  |  |  |
| **Player 24/1 *** | WD (n = 13) | CM (n = 4) |  |  |
| *Total distance [km]* | 10.34 ± 0.48 | 10.98 ± 0.38 | p = 0.01 | 1.48 |
| *High-intensity distance [km]* | 1.26 ± 0.18 | 1.40 ± 0.16 | p = 0.16 | 0.85 |
| *Sprinting distance [km]* | 0.40 ± 0.10 | 0.24 ± 0.05 | p < 0.01 | 1.85 |
| *accelerations* | 506 ± 80 | 510 ± 31 | p = 0.40 | 0.06 |
|  |  |  |  |  |
| **Player 24/2 ***** | WD (n = 13) | WB (n = 4) |  |  |
| *Total distance [km]* | 10.34 ± 0.48 | 10.96 ± 0.75 | p = 0.30 | 1.21 |
| *High-intensity distance [km]* | 1.26 ± 0.18 | 1.48 ± 0.23 | p < 0.01 | 1.23 |
| *Sprinting distance [km]* | 0.40 ± 0.1 | 0.37 ± 0.05 | p = 0.35 | 0.35 |
| *accelerations* | 506 ± 80 | 512 ± 39 | p = 0.37 | 0.09 |
|  |  |  |  |  |
| **Player 24/3 **** | WB (n = 4) | CM (n = 4) |  |  |
| *Total distance [km]* | 10.96 ± 0.75 | 10.98 ± 0.38 | p = 0.80 | 0.04 |
| *High-intensity distance [km]* | 1.48 ± 0.23 | 1.4 ± 0.16 | p = 0.49 | 0.47 |
| *Sprinting distance [km]* | 0.37 ± 0.05 | 0.24 ± 0.05 | p < 0.01 | 3.00 |
| *accelerations* | 512 ± 39 | 510 ± 31 | p > 0.99 | 0.07 |
|  |  |  |  |  |
| **Player 25/1 **** | WD (n = 5) | WB (n = 9) |  |  |
| *Total distance [km]* | 10.40 ± 0.57 | 10.45 ± 0.52 | p > 0.99 | 0.10 |
| *High-intensity distance [km]* | 1.42 ± 0.20 | 1.39 ± 0.16 | p > 0.99 | 0.19 |
| *Sprinting distance [km]* | 0.70 ± 0.04 | 0.72 ± 0.08 | p = 0.70 | 0.16 |
| *accelerations* | 504 ± 25 | 504 ± 33 | p > 0.99 | 0.00 |
|  |  |  |  |  |
| **Player 25/2 ***** | WD (n = 5) | WM (n = 4) |  |  |
| *Total distance [km]* | 10.40 ± 0.57 | 10.98 ± 0.76 | p = 0.13 | 1.00 |
| *High-intensity distance [km]* | 1.42 ± 0.20 | 1.56 ± 0.21 | p = 0.38 | 0.78 |
| *Sprinting distance [km]* | 0.70 ± 0.04 | 0.67 ± 0.12 | p = 0.83 | 0.40 |
| *accelerations* | 504 ± 25 | 524 ± 13 | p = 0.14 | 1.09 |
|  |  |  |  |  |
| **Player 25/3 ***** | WB (n = 9) | WM (n = 4) |  |  |
| *Total distance [km]* | 10.45 ± 0.52 | 10.98 ± 0.76 | p = 0.12 | 0.97 |
| *High-intensity distance [km]* | 1.39 ± 0.16 | 1.56 ± 0.21 | p = 0.13 | 1.06 |
| *Sprinting distance [km]* | 0.72 ± 0.08 | 0.67 ± 0.12 | p = 0.58 | 0.59 |
| *accelerations* | 504 ± 33 | 524 ± 13 | p = 0.09 | 0.75 |

ES – Effect size; 95% CI – 95% Confidence interval; CD – Central defender; WD – Wide defender; WB – Wing back; CM – Central midfielder; WM – Wide midfielder; FW – Forward; * – large effect size differences between positions for at least three performance parameters; ** large effect size differences between positions for a maximum of one performance parameter; *** large effect size differences between positions for two performance parameters and trivial-to-moderate effect size differences for two performance parameters
